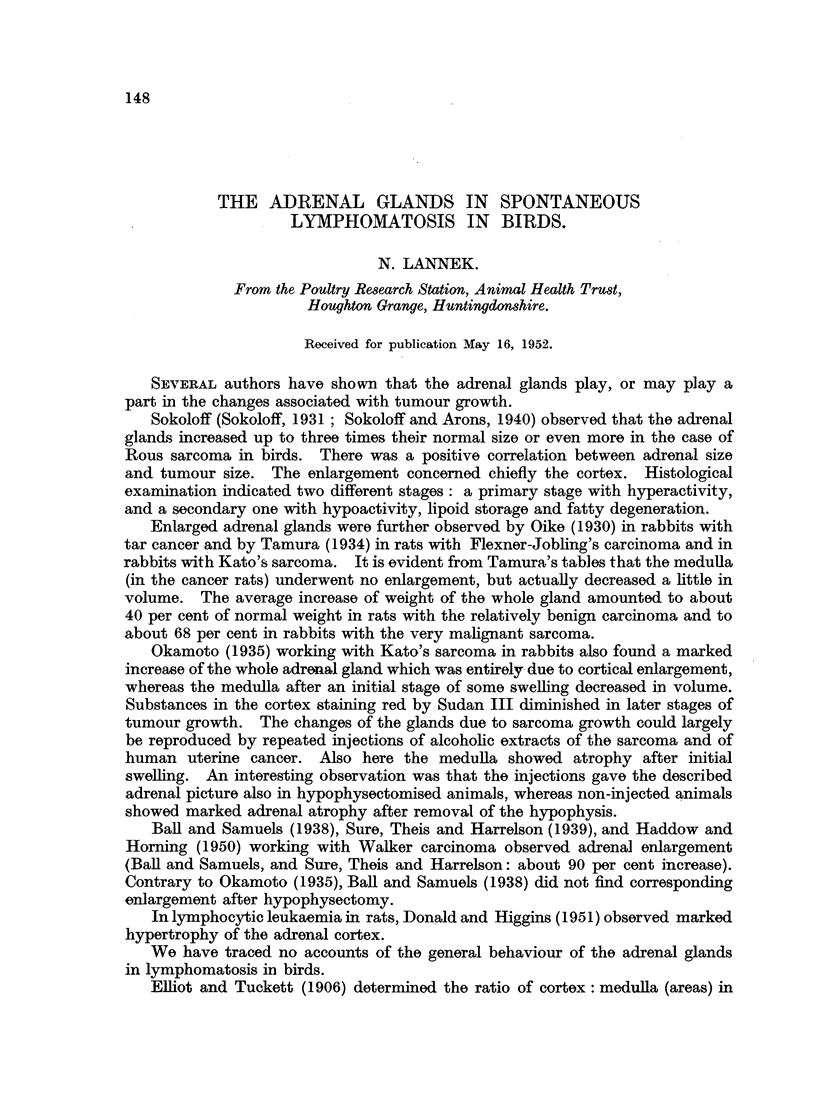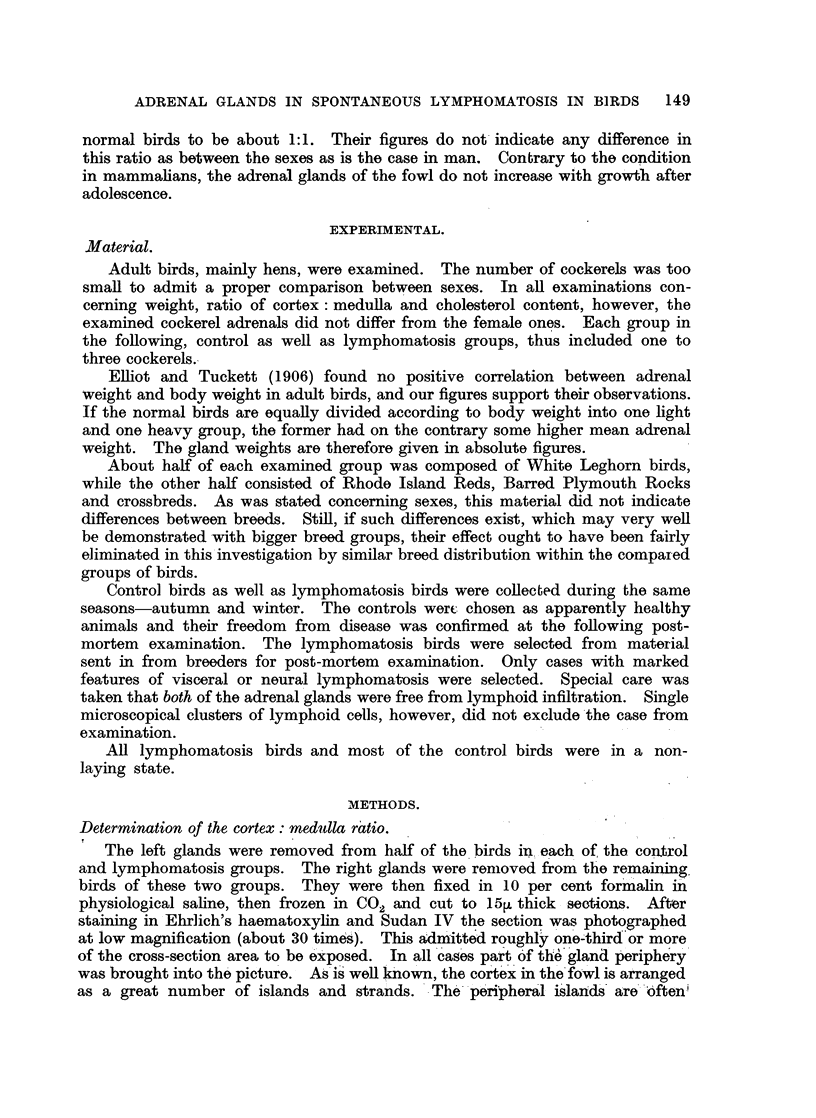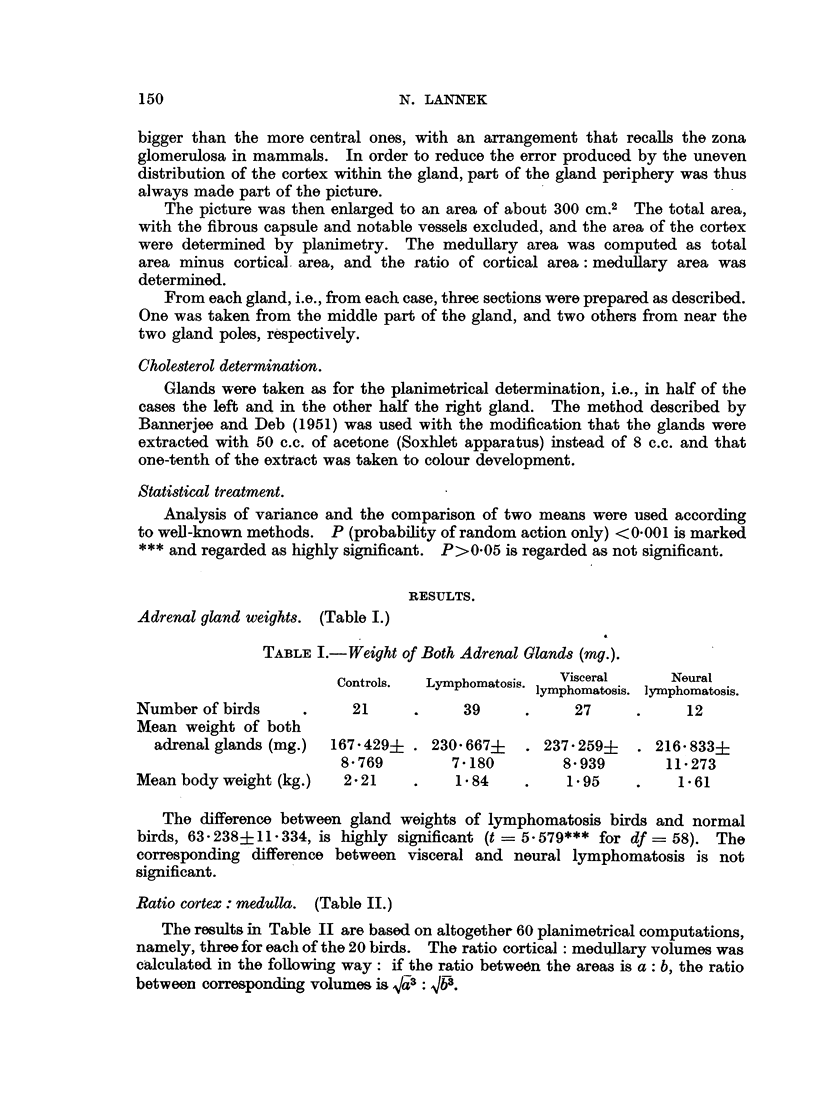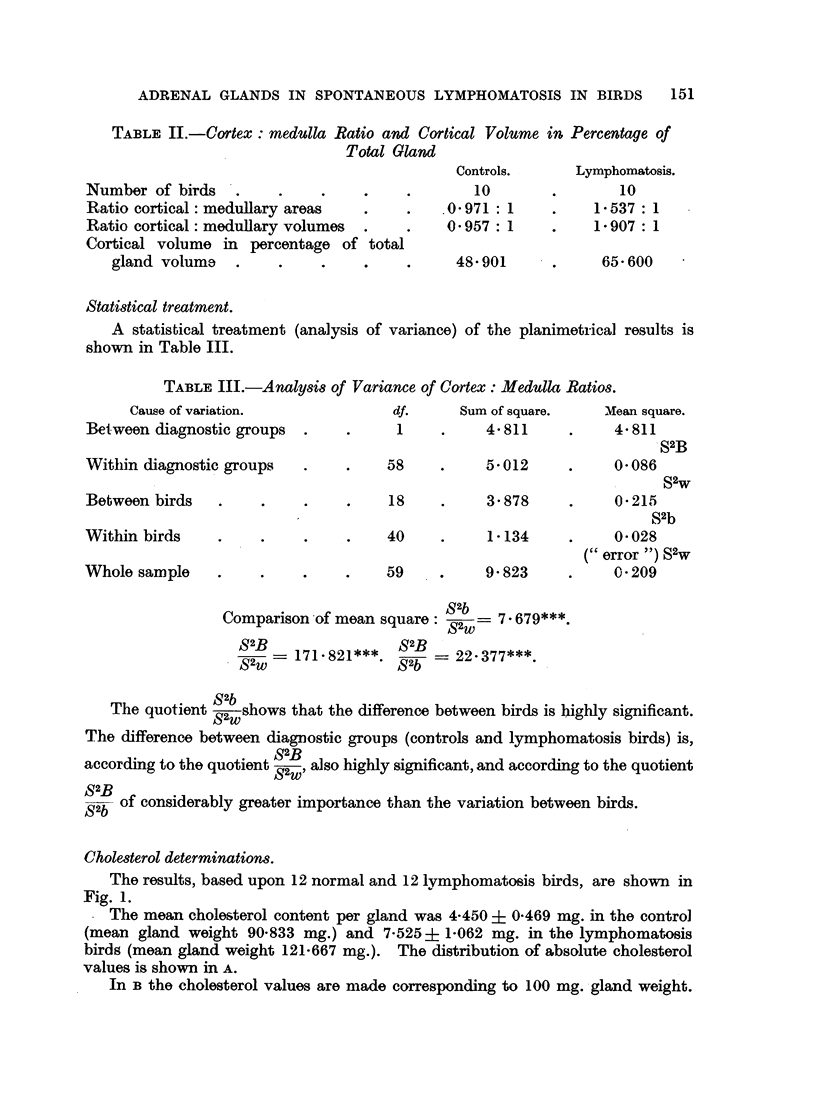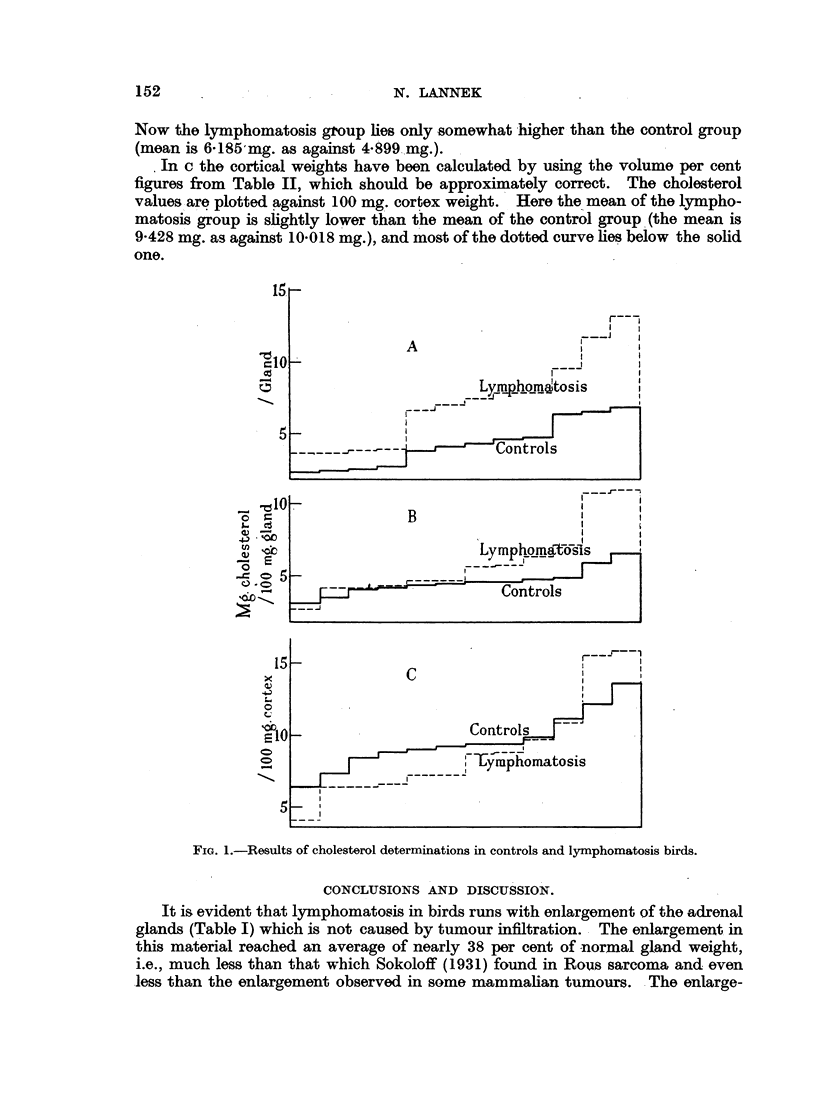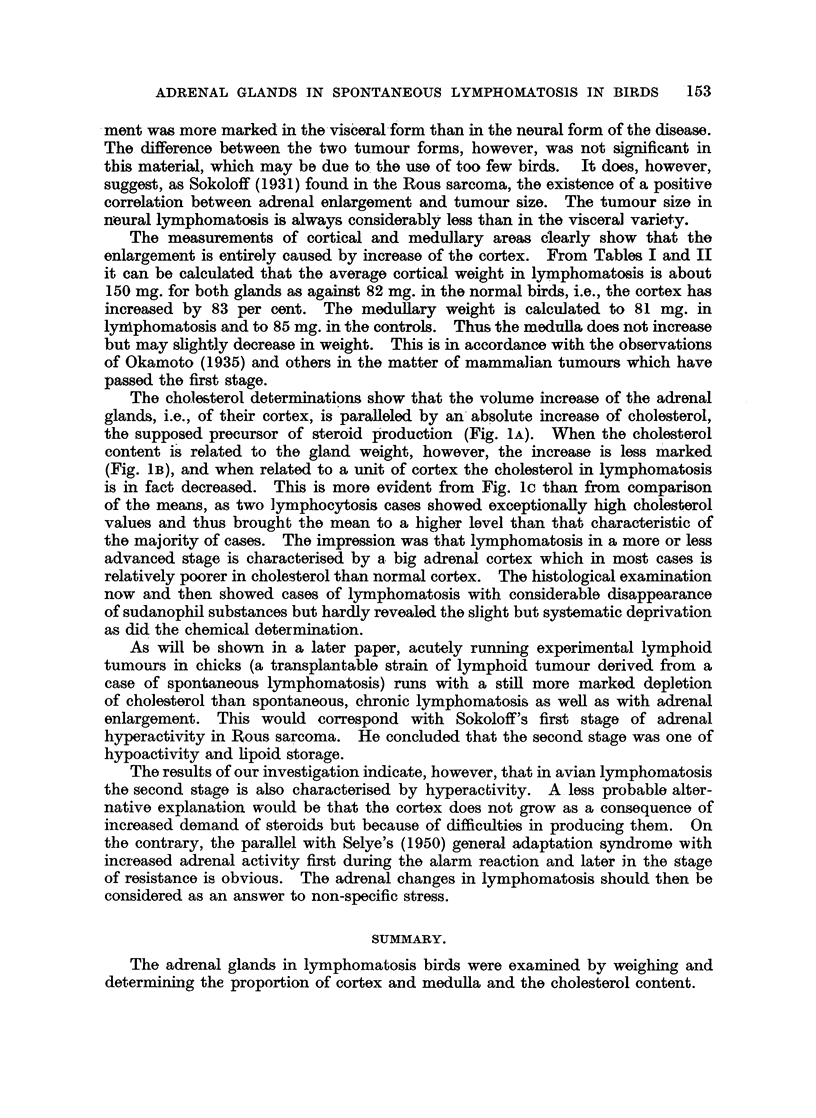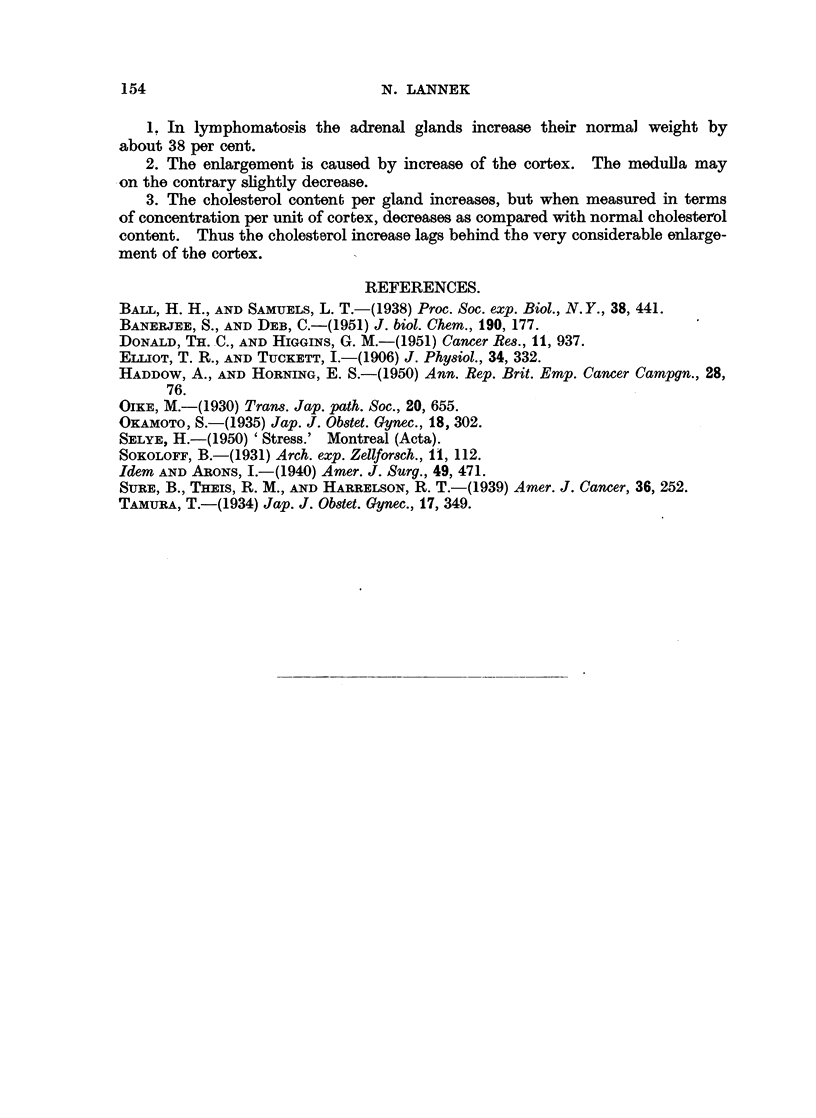# The Adrenal Glands in Spontaneous Lymphomatosis in Birds

**DOI:** 10.1038/bjc.1952.17

**Published:** 1952-06

**Authors:** N. Lannek


### THE ADRENAL GLANDS IN SPONTANEOUS LYMPHOMATOSIS IN BIRDS.

N. LANNEK.

*From the Poultry Research Station, Animal Health Trust, Houghton Grange, Huntingdonshire.*

Received for publication May 16, 1952.

SEVERAL authors have shown that the adrenal glands play, or may play a part in the changes associated with tumour growth.

Sokoloff (Sokoloff, 1931 ; Sokoloff and Arons, 1940) observed that the adrenal glands increased up to three times their normal size or even more in the case of Rous sarcoma in birds. There was a positive correlation between adrenal size and tumour size. The enlargement concerned chiefly the cortex. Histological examination indicated two different stages : a primary stage with hyperactivity, and a secondary one with hypoactivity, lipoid storage and fatty degeneration.

Enlarged adrenal glands were further observed by Oike (1930) in rabbits with tar cancer and by Tamura (1934) in rats with Flexner-Jobling's carcinoma and in rabbits with Kato's sarcoma. It is evident from Tamura's tables that the medulla (in the cancer rats) underwent no enlargement, but actually decreased a little in volume. The average increase of weight of the whole gland amounted to about 40 per cent of normal weight in rats with the relatively benign carcinoma and to about 68 per cent in rabbits with the very malignant sarcoma.

Okamoto (1935) working with Kato's sarcoma in rabbits also found a marked increase of the whole adrenal gland which was entirely due to cortical enlargement, whereas the medulla after an initial stage of some swelling decreased in volume. Substances in the cortex staining red by Sudan III diminished in later stages of tumour growth. The changes of the glands due to sarcoma growth could largely be reproduced by repeated injections of alcoholic extracts of the sarcoma and of human uterine cancer. Also here the medulla showed atrophy after initial swelling. An interesting observation was that the injections gave the described adrenal picture also in hypophysectomised animals, whereas non-injected animals showed marked adrenal atrophy after removal of the hypophysis.

Ball and Samuels (1938), Sure, Theis and Harrelson (1939), and Haddow and Horning (1950) working with Walker carcinoma observed adrenal enlargement (Ball and Samuels, and Sure, Theis and Harrelson: about 90 per cent increase). Contrary to Okamoto (1935), Ball and Samuels (1938) did not find corresponding enlargement after hypophysectomy.

In lymphocytic leukaemia in rats, Donald and Higgins (1951) observed marked hypertrophy of the adrenal cortex.

We have traced no accounts of the general behaviour of the adrenal glands in lymphomatosis in birds.

Elliot and Tuckett (1906) determined the ratio of cortex : medulla (areas) in normal birds to be about 1:1. Their figures do not indicate any difference in this ratio as between the sexes as is the case in man. Contrary to the condition in mammalians, the adrenal glands of the fowl do not increase with growth after adolescence.

### EXPERIMENTAL.

### *Material.*

Adult birds, mainly hens, were examined. The number of cockerels was too small to admit a proper comparison between sexes. In all examinations concerning weight, ratio of cortex : medulla and cholesterol content, however, the examined cockerel adrenals did not differ from the female ones. Each group in the following, control as well as lymphomatosis groups, thus included one to three cockerels.

Elliot and Tuckett (1906) found no positive correlation between adrenal weight and body weight in adult birds, and our figures support their observations. If the normal birds are equally divided according to body weight into one light and one heavy group, the former had on the contrary some higher mean adrenal weight. The gland weights are therefore given in absolute figures.

About half of each examined group was composed of White Leghorn birds, while the other half consisted of Rhode Island Reds, Barred Plymouth Rocks and crossbreds. As was stated concerning sexes, this material did not indicate differences between breeds. Still, if such differences exist, which may very well be demonstrated with bigger breed groups, their effect ought to have been fairly eliminated in this investigation by similar breed distribution within the compared groups of birds.

Control birds as well as lymphomatosis birds were collected during the same seasons—autumn and winter. The controls were chosen as apparently healthy animals and their freedom from disease was confirmed at the following post-mortem examination. The lymphomatosis birds were selected from material sent in from breeders for post-mortem examination. Only cases with marked features of visceral or neural lymphomatosis were selected. Special care was taken that *both* of the adrenal glands were free from lymphoid infiltration. Single microscopical clusters of lymphoid cells, however, did not exclude the case from examination.

All lymphomatosis birds and most of the control birds were in a non-laying state.

### METHODS.

### *Determination of the cortex : medulla ratio.*

The left glands were removed from half of the birds in each of the control and lymphomatosis groups. The right glands were removed from the remaining birds of these two groups. They were then fixed in 10 per cent formalin in physiological saline, then frozen in $CO_2$ and cut to 15$\mu$ thick sections. After staining in Ehrlich's haematoxylin and Sudan IV the section was photographed at low magnification (about 30 times). This admitted roughly one-third or more of the cross-section area to be exposed. In all cases part of the gland periphery was brought into the picture. As is well known, the cortex in the fowl is arranged as a great number of islands and strands. The peripheral islands are often bigger than the more central ones, with an arrangement that recalls the zona glomerulosa in mammals. In order to reduce the error produced by the uneven distribution of the cortex within the gland, part of the gland periphery was thus always made part of the picture.

The picture was then enlarged to an area of about 300 cm.$^2$ The total area, with the fibrous capsule and notable vessels excluded, and the area of the cortex were determined by planimetry. The medullary area was computed as total area minus cortical area, and the ratio of cortical area : medullary area was determined.

From each gland, i.e., from each case, three sections were prepared as described. One was taken from the middle part of the gland, and two others from near the two gland poles, respectively.

*Cholesterol determination.*

Glands were taken as for the planimetrical determination, i.e., in half of the cases the left and in the other half the right gland. The method described by Bannerjee and Deb (1951) was used with the modification that the glands were extracted with 50 c.c. of acetone (Soxhlet apparatus) instead of 8 c.c. and that one-tenth of the extract was taken to colour development.

*Statistical treatment.*

Analysis of variance and the comparison of two means were used according to well-known methods. $P$ (probability of random action only) $<0{\cdot}001$ is marked *** and regarded as highly significant. $P>0{\cdot}05$ is regarded as not significant.

### RESULTS.

*Adrenal gland weights.* (Table I.)

TABLE I.—*Weight of Both Adrenal Glands (mg.).*

| | Controls. | Lymphomatosis. | Visceral lymphomatosis. | Neural lymphomatosis. |
|---|---|---|---|---|
| Number of birds | 21 | 39 | 27 | 12 |
| Mean weight of both adrenal glands (mg.) | 167·429± 8·769 | 230·667± 7·180 | 237·259± 8·939 | 216·833± 11·273 |
| Mean body weight (kg.) | 2·21 | 1·84 | 1·95 | 1·61 |

The difference between gland weights of lymphomatosis birds and normal birds, 63·238±11·334, is highly significant ($t = 5{\cdot}579$*** for $df = 58$). The corresponding difference between visceral and neural lymphomatosis is not significant.

*Ratio cortex : medulla.* (Table II.)

The results in Table II are based on altogether 60 planimetrical computations, namely, three for each of the 20 birds. The ratio cortical : medullary volumes was calculated in the following way: if the ratio between the areas is $a : b$, the ratio between corresponding volumes is $\sqrt{a^3} : \sqrt{b^3}$.

TABLE II.—*Cortex : medulla Ratio and Cortical Volume in Percentage of Total Gland*

| | Controls. | Lymphomatosis. |
|---|---|---|
| Number of birds | 10 | 10 |
| Ratio cortical : medullary areas | 0·971 : 1 | 1·537 : 1 |
| Ratio cortical : medullary volumes | 0·957 : 1 | 1·907 : 1 |
| Cortical volume in percentage of total gland volume | 48·901 | 65·600 |

*Statistical treatment.*

A statistical treatment (analysis of variance) of the planimetrical results is shown in Table III.

TABLE III.—*Analysis of Variance of Cortex : Medulla Ratios.*

| Cause of variation. | *df.* | Sum of square. | Mean square. |
|---|---|---|---|
| Between diagnostic groups | 1 | 4·811 | 4·811 $S^2B$ |
| Within diagnostic groups | 58 | 5·012 | 0·086 $S^2w$ |
| Between birds | 18 | 3·878 | 0·215 $S^2b$ |
| Within birds | 40 | 1·134 | 0·028 ("error") $S^2w$ |
| Whole sample | 59 | 9·823 | 0·209 |

$$\text{Comparison of mean square : } \frac{S^2b}{S^2w} = 7\cdot679^{***}.$$

$$\frac{S^2B}{S^2w} = 171\cdot821^{***}. \quad \frac{S^2B}{S^2b} = 22\cdot377^{***}.$$

The quotient $\frac{S^2b}{S^2w}$ shows that the difference between birds is highly significant. The difference between diagnostic groups (controls and lymphomatosis birds) is, according to the quotient $\frac{S^2B}{S^2w}$, also highly significant, and according to the quotient $\frac{S^2B}{S^2b}$ of considerably greater importance than the variation between birds.

*Cholesterol determinations.*

The results, based upon 12 normal and 12 lymphomatosis birds, are shown in Fig. 1.

The mean cholesterol content per gland was 4·450 ± 0·469 mg. in the control (mean gland weight 90·833 mg.) and 7·525 ± 1·062 mg. in the lymphomatosis birds (mean gland weight 121·667 mg.). The distribution of absolute cholesterol values is shown in A.

In B the cholesterol values are made corresponding to 100 mg. gland weight.

Now the lymphomatosis group lies only somewhat higher than the control group (mean is 6·185 mg. as against 4·899 mg.).

In C the cortical weights have been calculated by using the volume per cent figures from Table II, which should be approximately correct. The cholesterol values are plotted against 100 mg. cortex weight. Here the mean of the lymphomatosis group is slightly lower than the mean of the control group (the mean is 9·428 mg. as against 10·018 mg.), and most of the dotted curve lies below the solid one.

FIG. 1.—Results of cholesterol determinations in controls and lymphomatosis birds.

### CONCLUSIONS AND DISCUSSION.

It is evident that lymphomatosis in birds runs with enlargement of the adrenal glands (Table I) which is not caused by tumour infiltration. The enlargement in this material reached an average of nearly 38 per cent of normal gland weight, i.e., much less than that which Sokoloff (1931) found in Rous sarcoma and even less than the enlargement observed in some mammalian tumours. The enlargement was more marked in the visceral form than in the neural form of the disease. The difference between the two tumour forms, however, was not significant in this material, which may be due to the use of too few birds. It does, however, suggest, as Sokoloff (1931) found in the Rous sarcoma, the existence of a positive correlation between adrenal enlargement and tumour size. The tumour size in neural lymphomatosis is always considerably less than in the visceral variety.

The measurements of cortical and medullary areas clearly show that the enlargement is entirely caused by increase of the cortex. From Tables I and II it can be calculated that the average cortical weight in lymphomatosis is about 150 mg. for both glands as against 82 mg. in the normal birds, i.e., the cortex has increased by 83 per cent. The medullary weight is calculated to 81 mg. in lymphomatosis and to 85 mg. in the controls. Thus the medulla does not increase but may slightly decrease in weight. This is in accordance with the observations of Okamoto (1935) and others in the matter of mammalian tumours which have passed the first stage.

The cholesterol determinations show that the volume increase of the adrenal glands, i.e., of their cortex, is paralleled by an absolute increase of cholesterol, the supposed precursor of steroid production (Fig. 1A). When the cholesterol content is related to the gland weight, however, the increase is less marked (Fig. 1B), and when related to a unit of cortex the cholesterol in lymphomatosis is in fact decreased. This is more evident from Fig. 1C than from comparison of the means, as two lymphocytosis cases showed exceptionally high cholesterol values and thus brought the mean to a higher level than that characteristic of the majority of cases. The impression was that lymphomatosis in a more or less advanced stage is characterised by a big adrenal cortex which in most cases is relatively poorer in cholesterol than normal cortex. The histological examination now and then showed cases of lymphomatosis with considerable disappearance of sudanophil substances but hardly revealed the slight but systematic deprivation as did the chemical determination.

As will be shown in a later paper, acutely running experimental lymphoid tumours in chicks (a transplantable strain of lymphoid tumour derived from a case of spontaneous lymphomatosis) runs with a still more marked depletion of cholesterol than spontaneous, chronic lymphomatosis as well as with adrenal enlargement. This would correspond with Sokoloff's first stage of adrenal hyperactivity in Rous sarcoma. He concluded that the second stage was one of hypoactivity and lipoid storage.

The results of our investigation indicate, however, that in avian lymphomatosis the second stage is also characterised by hyperactivity. A less probable alternative explanation would be that the cortex does not grow as a consequence of increased demand of steroids but because of difficulties in producing them. On the contrary, the parallel with Selye's (1950) general adaptation syndrome with increased adrenal activity first during the alarm reaction and later in the stage of resistance is obvious. The adrenal changes in lymphomatosis should then be considered as an answer to non-specific stress.

### SUMMARY.

The adrenal glands in lymphomatosis birds were examined by weighing and determining the proportion of cortex and medulla and the cholesterol content.

1. In lymphomatosis the adrenal glands increase their normal weight by about 38 per cent.

2. The enlargement is caused by increase of the cortex. The medulla may on the contrary slightly decrease.

3. The cholesterol content per gland increases, but when measured in terms of concentration per unit of cortex, decreases as compared with normal cholesterol content. Thus the cholesterol increase lags behind the very considerable enlargement of the cortex.

### REFERENCES.

Ball, H. H., and Samuels, L. T.—(1938) *Proc. Soc. exp. Biol., N.Y.*, **38**, 441.
Banerjee, S., and Deb, C.—(1951) *J. biol. Chem.*, **190**, 177.
Donald, Th. C., and Higgins, G. M.—(1951) *Cancer Res.*, **11**, 937.
Elliot, T. R., and Tuckett, I.—(1906) *J. Physiol.*, **34**, 332.
Haddow, A., and Horning, E. S.—(1950) *Ann. Rep. Brit. Emp. Cancer Campgn.*, **28**, 76.
Oike, M.—(1930) *Trans. Jap. path. Soc.*, **20**, 655.
Okamoto, S.—(1935) *Jap. J. Obstet. Gynec.*, **18**, 302.
Selye, H.—(1950) 'Stress.' Montreal (Acta).
Sokoloff, B.—(1931) *Arch. exp. Zellforsch.*, **11**, 112.
*Idem* and Arons, I.—(1940) *Amer. J. Surg.*, **49**, 471.
Sure, B., Theis, R. M., and Harrelson, R. T.—(1939) *Amer. J. Cancer*, **36**, 252.
Tamura, T.—(1934) *Jap. J. Obstet. Gynec.*, **17**, 349.